# 2′-Fluorinated nucleoside chemistry for new drug discovery: achievements and prospects

**DOI:** 10.1093/nsr/nwae331

**Published:** 2024-10-01

**Authors:** Yonggang Meng, Nannan Sun, Lan Liang, Bin Yu, Junbiao Chang

**Affiliations:** College of Chemistry, Pingyuan Laboratory, State Key Laboratory of Antiviral Drugs, Zhengzhou University, Zhengzhou 450001, China; School of Pharmaceutical Sciences, Zhengzhou University, Zhengzhou 450001, China; School of Pharmaceutical Sciences, Zhengzhou University, Zhengzhou 450001, China; State Key Laboratory of Antiviral Drugs, School of Chemistry and Chemical Engineering, Henan Normal University, Xinxiang 453007, China; College of Chemistry, Pingyuan Laboratory, State Key Laboratory of Antiviral Drugs, Zhengzhou University, Zhengzhou 450001, China; College of Chemistry, Pingyuan Laboratory, State Key Laboratory of Antiviral Drugs, Zhengzhou University, Zhengzhou 450001, China; School of Pharmaceutical Sciences, Zhengzhou University, Zhengzhou 450001, China; State Key Laboratory of Antiviral Drugs, School of Chemistry and Chemical Engineering, Henan Normal University, Xinxiang 453007, China

**Keywords:** fluorinated nucleosides, antitumor activity, antiviral activity, drug discovery

## Abstract

Fluorinated nucleosides are an important class of modified nucleosides that have demonstrated therapeutic potential for treating various human diseases, especially viral infections and cancer. Many fluorinated nucleosides have advanced into clinical trials or have been approved by the FDA for use in patients. Among these fluorinated nucleosides, azvudine, developed by us, has been officially approved by the National Medical Products Administration for the treatment of coronavirus disease 2019 (COVID-19) and human immunodeficiency virus, indicating the therapeutic promise of fluorinated nucleosides. In view of the therapeutic promise of fluorinated nucleosides for antiviral and anticancer therapy, in this Review we will provide a comprehensive overview of well-established 2′-fluorinated nucleosides approved for use in the market or those in clinical stages for antiviral and antitumor therapies, highlighting the drug discovery strategies, structure-activity relationship studies, mechanisms of action, and preclinical/clinical studies and also discuss the challenges and future directions for nucleoside-based new drug discovery.

## Teaser

This review showcases the journey of 2′-fluorinated nucleosides from bench to clinic for antiviral and antitumor therapies, highlighting the drug discovery strategies, SAR studies, mechanisms of actions, and preclinical/clinical studies.

## INRODUCTION

Nucleoside analogs (NAs) show promise in treating cancer and viral infections such as hepatitis B virus (HBV), herpes simplex virus (HSV), human immunodeficiency virus (HIV), severe acute respiratory syndrome coronavirus 2 (SARS-CoV-2), and hepatitis C virus (HCV), etc. [1–4]. Breakthroughs have been achieved on the discovery of NAs by structural modifications on nucleobase, ribose ring, and the nucleic acid backbone. Chemically modified nucleoside and nucleotide analogs mimic their natural counterparts, but with enhanced pharmacokinetic (PK) and pharmacodynamic (PD) properties to improve their biological activity [5,6].

About 20%–25% of drugs or candidates contain the fluorine atom. Introducing fluorine atom(s) into NAs is a feasible way to modulate physicochemical, adsorption, and distribution properties [7]. Fluorine substitution is believed to provide several benefits, including: (1) having the same number of electrons in the outermost layer with the OH moiety and similar size to hydrogen (H) atom, the fluorine atom is usually used as the pharmacophore bioisostere of H and OH, affecting the binding affinity [8]. (2) The electronegativity of the fluorine atom is greater than that of the hydrogen atom, and thus the C–F bond strength is stronger than that of the C–H bond. Therefore, fluorinated nucleosides and nucleotides can modify electronic properties and exhibit enhanced biophysical and biochemical characteristics [8]. (3) As a strong electronegative element, fluorine is also a good hydrogen-bond acceptor. The C–F bond length (1.35 Å) is close to that of the C–O bond (1.43 Å), hence the C–F bond is an ideal isopolar and isosteric alternative of the C–O bond. (4) Fluoro substitution could influence drug lipophilicity. Fluorinated compounds often have improved lipophilicity (logP), which associates with enhanced membrane permeability and cellular penetration, forming hydrophobic interactions with specific binding sites of target proteins [9]. (5) Because of the high electronegativity of fluorine, the electron-withdrawing effect of fluorine substitution cannot be ignored [9]. Fluorine substitution affects the acid/base (pKa) properties of neighboring functional groups, for example, induced by the fluorine atom(s), the amine pKa would be lowered with weakened molecular alkalescency.

**Figure 1.**
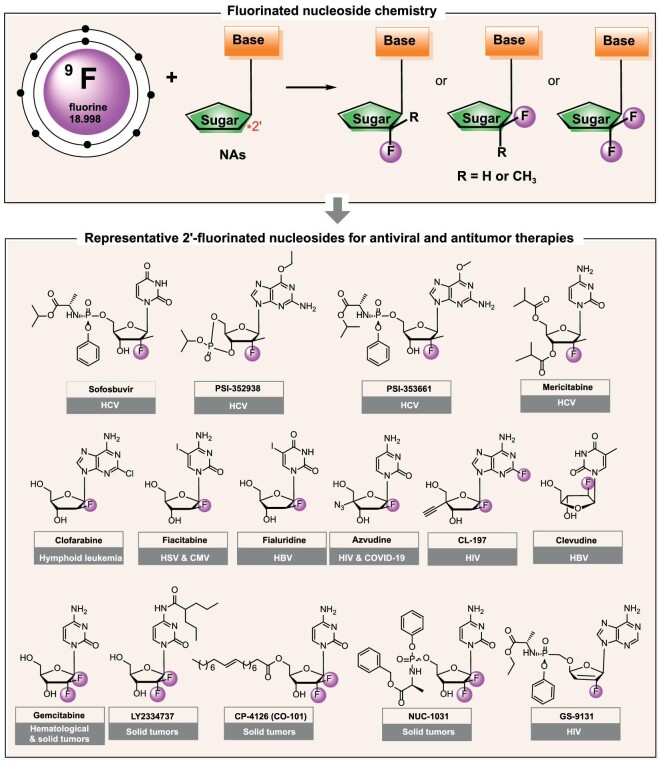
Representative 2′-fluorinated nucleosides approved or in clinical trials.

The role of the substituent on the 2′-carbon atom of nucleic acids inspired the development of 2′-C modified nucleosides. Because of having the same number of electrons in the outermost layer with –OH and similar size to –H, the fluorine atom is usually used as the pharmacophore bioisostere of H and OH. Introducing a fluorine substituent at the 2′-position alters the stereo-electronic properties of sugar and fixes the conformation of fluorinated nucleosides depending on the configuration. The C-2′ fluorinated nucleosides are well studied. Until now, more than ten 2′-fluorinated nucleosides have progressed into clinical studies or been approved as anticancer and antiviral drugs. Notably, the 2′-fluorinated nucleoside drug azvudine, developed by us, has been approved for treating COVID-19 and HIV infections, and another 2′-β-fluoroadenosine CL-197 has also advanced into clinical stage for treating HIV-1 infection (registration number: CXHL2200529), indicating the therapeutic promise of fluorinated nucleosides [10]. In this Review, we aim to provide an overview of 2′-fluorinated nucleosides available in the market or at clinical stages for antiviral and antitumor therapies (Fig. 1). We will highlight drug discovery strategies, structure-activity relationship (SAR) studies, different mechanisms of action, as well as preclinical and clinical studies. Additionally, we will share our perspectives on fluorinated nucleosides.

## NUCLEOSIDES CONTAINING A FLUORINE ATOM AT C-2′-DOWN (2′-DEOXY-2′-α-FLUORO-2′-β-C-METHYL NUCLEOSIDES)

**Figure 2.**
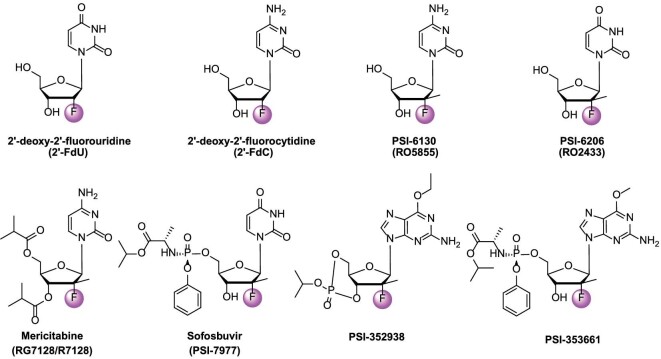
The structures of 2′-FdU, 2′-FdC, PSI-6130, and PSI-6202 as well as the prodrugs mericitabine, sofosbuvir, PSI-352938, and PSI-353661.

In 1961, Codington *et al*. synthesized the first 2′-fluoro nucleoside (2′-deoxy-2′-fluorouridine, 2′-FdU, Fig. 2) [11]. Subsequent studies showed that 2′-fluoro nucleosides were stable against degradation by nucleases [12], then a series of 2′-deoxy-2′-fluoro nucleosides with different nucleobases were synthesized. Among these compounds, 2′-deoxy-2′-fluorocytidine (2′-FdC, Fig. 2) stood out with a varied degree of antiviral and antitumor activities [13,14]. 2′-FdC reduced HCV replicon RNA levels with a 90% effective concentration (EC_90_) of 5.0 μM, and showed low cytotoxicity with a 50% cytotoxic concentration (CC_50_) value above 100 μM [13]. It was also found to have marginally antiviral activity against SARS-CoV-2 with an EC_50_ of 175.2 μM [15]. Because of the poor selectivity and cytostasis-induced effect at the EC_90_ value of 2′-FdC [16], the SAR studies were carried out mainly by introducing substituents on the aromatic nucleoside base and 2′-carbon atom of nucleic acids based on 2′-FdC.

Scientists at Pharmasset, Inc. obtained *β*-*D*-2′-deoxy-2′-fluoro-2′-C-methylcytidine (PSI-6130, Fig. 2) by a diethylaminosulfur trifluoride (DAST) fluorination, and PSI-6130 was identified to possess highly specific HCV replicon inhibition activity targeting NS5B polymerase. PSI-6130 was much more potent and safer than 2′-FdC, and no cytotoxic effects were observed on human bone marrow, peripheral blood mononuclear cells (PBMCs), or mitochondria [14,17]. However, PSI-6130, catalyzed by human cytidine deaminase, was easily converted to an inactive PSI-6206 (RO2433, Fig. 2) [18]. Single-dose PK studies of PSI-6130 in rhesus monkeys indicated a relatively low oral bioavailability (*F* = 24.0%), and the deamination of PSI-6130 cannot be neglected [19]. In order to ameliorate the PK properties, the prodrug strategy was employed to endow PSI-6130 prodrugs with higher *in vivo* anti-HCV efficiency and improved membrane permeability, stability, distribution, etc. Sofosbuvir, mericitabine, PSI-352938, and PSI-353661 are representative compounds (Fig. 2).

### Mericitabine

**Figure 3.**
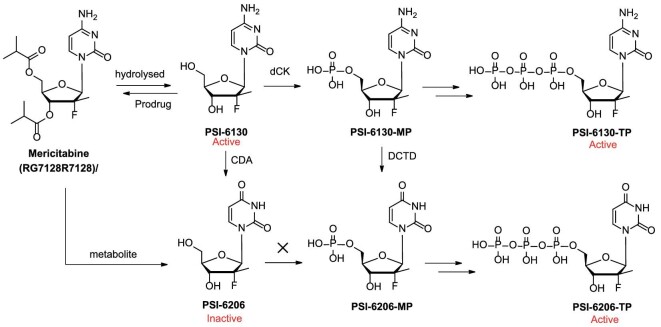
The bioactive metabolites of mericitabine.

The prodrug strategy, by capping the 3′,5′-hydroxyl groups of the nucleoside and/or the C-4 amino group, was employed to improve oral bioavailability and reduce PSI-6130 deamination for HCV treatment. The 3′- and 5′-hydroxyls were converted to the corresponding esters, carbamates, and carbonates, and the C-4 amino group was protected as carbamates, ureas, amides, and imines [20]. Of all these prodrugs, the 3′,5′-diisobutyryl ester prodrug mericitabine (RG7128/R7128, Fig. 3) was prepared from PSI-6130 and isobutyryl chloride [21]. Mericitabine potently inhibited stable and transient replicons with the EC_50_ values comparable to those of PSI-6130 (Table 1). The Phase I clinical study of mericitabine (500 mg or 1000 mg twice daily) in combination with the NS3/4A protease inhibitor danoprevir (100 mg or 200 mg every 8 h or 600 mg or 900 mg twice daily) showed that the combination treatment decreased the viral load significantly and rapidly [22], the median change in HCV RNA concentration ranged from −4.9 to −5.1 log10 IU/mL without treatment-related serious adverse events (ClinicalTrials.gov identifier: NCT00801255). In a Phase II clinical study, mericitabine showed strong antiviral effects in HCV patients. No resistance was detected with a 1500 mg dose taken twice daily after two weeks of monotherapy or with doses of 1000 mg and 1500 mg twice daily following four weeks of combined treatment with the standard care (interferon (IFN)/ribavirin (RBV)) [23]. Though the prodrug strategy improved the oral bioavailability of PSI-6130, the clinical data of mericitabine demonstrated its efficacy as an HCV inhibitor, apart from the active cytidine metabolite PSI-6130, mericitabine also formed the inactive uridine metabolite (PSI-6206, Fig. 3). Interestingly, the nucleoside PSI-6206 was inactive, but its triphosphate (PSI-6206-TP) was a potent inhibitor of HCV NS5B and S282T NS5B (with *K*_i_ of 0.42 μM and 22 μM, respectively). Besides, PSI-6206-TP was much more stable than PSI-6130-TP in primary human hepatocytes. PSI-6130-TP was detected with steady-state levels at 24–48 h, while the steady-state levels of the uridine congener PSI-6206-TP were 48–72 h [18]. The activity of PSI-6206-TP and its long half-life characteristics indicated a possibility of developing anti-HCV agents that could be dosed once daily. But the direct phosphorylation of PSI-6206 to PSI-6206-TP was limited because of the inhibition of monophosphate by nucleoside kinase [18,24]. The formation of the 5′-triphosphate of PSI-6206 requires the deamination of PSI-6130-MP catalyzed by deoxycytidylate deaminase, then PSI-6206-MP was subsequently phosphorylated to its diphosphate and triphosphate by cellular uridine/cytidine monophosphate kinase (UMP/CMP) and nucleoside diphosphate kinase, respectively (Fig. 3). The PSI-6206-TP metabolic pathway indicated that the uridine monophosphate could be developed as an ideal oral direct-acting antiviral by delivering PSI-6206-MP or its derivatives into the body, particularly the liver. These findings enlightened the discovery of anti-HCV drugs.

**Table 1.** Comparison of pharmacological and pharmacokinetic properties of mericitabine and nucleoside PSI-6130

						Rat PK (10 mg/kg)	
Compound	EC_50_ (μM), CloneA	CC_50_ (μM)	Stability SGF (pH 1.2), *t*_1/2_ (h), 37^o^C	Stability SIF (pH 7.4), *t*_1/2_ (h), 37^o^C	Caco2 Papp*10^−6^ (cm/s)	AUC_0–24_ (μM/mL·h)	*C*_max_ (μg/mL)
PSI-6130	3.03	>100	>20	>20	0.21	2.97	0.6
mericitabine	2.5	>100	25	36	6.4	16.17	1.86

### Sofosbuvir

In 2010, scientists at Pharmasset, Inc. reported a series of phosphoramidate prodrugs that can bypass the nonproductive phosphorylation step and deliver PSI-6206-MP directly into liver. They explored the carboxylic acid ester group (R^1^), the R^2^ group and the R^3^ phosphate ester group [25]. The R^2^ group was the less steric alkyl group, while the R^3^ group was the mono- or dihalogenated phenyl ring. The compounds substituted with the simple or branched alkyl groups (R^1^) showed submicromolar activity. When R^1^ was the relatively large group such as *n*-butyl, 2-butyl, *n*-pentyl or Bn, cytotoxicity was observed for the compounds. As shown in Table 2, PSI-7672, PSI-7851 and PSI-8118 showed submicromolar activity against HCV. PSI-8118 was a potent HCV inhibitor with an EC_90_ of 0.04 μM, but showed some cytotoxicity. PSI-7851 showed good activity, low cytotoxicity, and acceptable stability. Besides, it demonstrated the best PK parameters in rat after an oral dose of 50 mg/kg (PSI-6206-TP maximum concentration (*C*_max_) and area under the plasma concentration-time curve from time 0 to last time of quantifiable concentration (AUC_(0–*t*)_) values were 1934 ng/g and 16 796 ng·h/g, respectively). *In vivo* PK studies also indicated that PSI-7851 was much more potent than PSI-7672 and PSI-8118 in dogs (*C*_max_ and AUC_(0–_*_t_*_)_ values were 6179 ng/g and 6894 ng·h/mL, respectively), and in cynomolgus monkeys (*C*_max_ and AUC_(0–_*_t_*_)_ values were 33 ng/g and 86 ng·h/mL, respectively). Two liver enzymes, carboxylesterase 1 and cathepsin A, are involved in the primary rate-limiting step of PSI-7851 metabolism, converting it into the parent compound PSI-6206-MP [26]. PSI-7851 is a mixture of diastereomers (1:1) at the phosphorus center of the phosphoramidate moiety and the (*S*)-P diastereomer (sofosbuvir/PSI-7977) was potent against most HCV genotypes, and well tolerated when given alone or used with ribavarin or pegylated IFN [27]. Clinical studies revealed that sofosbuvir was a potent anti-HCV agent with rapid virological response along (in 88%–94% of patients along with a 5.1–5.3 log_10_ decrease in viral load) with ideal PK properties. Sofosbuvir was rapidly converted to the mono- and triphosphate forms (PSI-6206-MP and PSI-6206-TP, respectively) in liver. There were no drug-related serious adverse events or discontinuations in these studies. Drug-drug interaction studies revealed that the combinations did not limit the use of sofosbuvir [28]. Because of good safety and efficacy, sofosbuvir (Sovaldi®) was approved by the FDA in December 2013 for treating HCV, quickly becoming a blockbuster drug. The following year, it also received approval from the European Medicines Agency. In 2015, the World Health Organization classified sofosbuvir as an essential medicine.

**Table 2.** The anti-HCV activity, cytotoxicity, and stability of PSI-7672, PSI-7851, and PSI-8118.

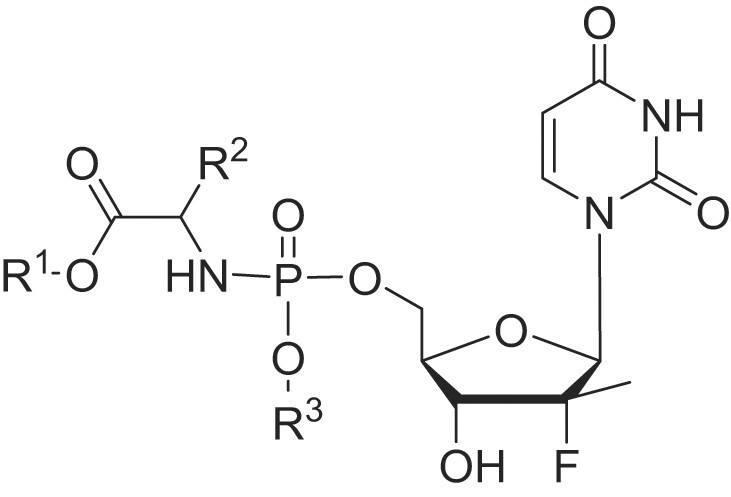									
						Stability *t*_1/2_ (h)			
Compd.	R^1^	R^2^	R^3^	CloneA EC_90_ (μM)	HepG2 CC_50_ (μM)	SGF	SIF	plasma	S9
PSI-7672	Me	Me	Ph	1.62	>100	15.5	>20	16.7	0.18
PSI-7851	*i*-Pr	Me	Ph	0.52	>100	22	>24	>24	0.57
PSI-8118	*c*-Hex	Me	4-F-Ph	0.04	70	20	>20	>24	0.18

### PSI-353661 and PSI-352938

**Figure 4.**
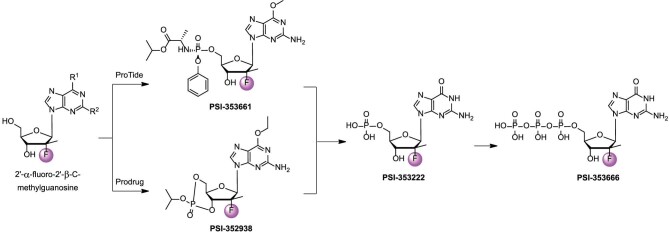
The discovery of PSI-353661, PSI-352938, and their metabolites.

Because of the inefficient uptake and/or conversion to active triphosphates, the purine nucleosides possessing the 2′-deoxy-2′-fluoro-2′-C-methylribofuranosyl moiety were reported to have moderate anti-HCV activities in the cell-based replicon assays [29]. Then, a series of *β*-*D*-2′-deoxy-2′-*α*-fluoro-2′-*β*-C-methylguanosine phosphoramidate prodrugs were found to inhibit HCV replication [30]. Relative to the parent guanosine analog, most of the prodrugs showed >1000-fold anti-HCV potency. PSI-353661 exhibited comparable anti-HCV activity against both wild-type and S282T resistant replicons (EC_90_ = 0.008 and 0.011 μM, respectively). In primary human hepatocytes, PSI-353661 was phosphorylated to the monophosphate intermediate PSI-353222, and then further to the active triphosphate PSI-352666 (Fig. 4). The triphosphate PSI-352666 rapidly reached its maximum concentration (>50 μM) at ∼4 h. Besides, PSI-353661 was non-toxic toward HepG2, BxPC-3, and CEM cells at 100 μM and also showed no mitochondrial toxicity [31]. PSI-353661 was selected as a preclinical candidate for HCV treatment.

In 2010, scientists at Pharmasset, Inc. disclosed another guanosine nucleoside prodrug containing a cyclic phosphate ester. SAR studies were performed at the 6-position of the base and the cyclic phosphate. Due to poor cell penetration, the unsubstituted guanosine methyl cyclic phosphate (6-OH) did not show acceptable potency. This finding was further confirmed by introducing the 6-alkoxy or alkyamine substituent and the compounds showed sub-micromolar anti-HCV activity. Both *cis-* and *trans-*isomers of 6-ethoxy isopropyl ester demonstrated desirable potency without significant cytotoxicity, the *cis-*isomer was thermodynamically more stable [32]. The *cis-*isomer PSI-352938 (GS-0938, Fig. 4) was further developed (EC_90_ = 1.37 μM, CC_50_ >100 μM) and showed no significant cellular or mitochondrial toxicity. Like the prodrug PSI-353661, PSI-352938 was converted to PSI-353666 and high levels of triphosphate were observed in both primary human hepatocytes [33]. In a rat PK study, significant exposure of PSI-352938 was observed in liver (*C*_max_ = 2829 ng/g, AUC_0–24_ = 13 234 ng·h/g at a single dose of 50 mg/kg) [32]. Clearance studies showed that PSI-352938 eradicated cells of HCV replicon RNA and prevented replicon rebound. Cross-resistance studies showcased that the replicons containing the NS5B S282T or the S96T/N142T alteration were sensitive to PSI-352938 [34], whereas the three amino acid changes (S15G/C223H/V321I) conferred a high level of resistance in genotype (GT)-2a replicons [35]. A Phase IIb study was conducted to assess the effectiveness and safety of PSI-352938 alone, in combination with sofosbuvir, or with both sofosbuvir and ribavirin. In 2013, the development of PSI-352938 was discontinued because of hepatic toxicity [36].

## NUCLEOSIDES CONTAINING A FLUORINE ATOM AT C-2′-UP (2′-DEOXY-2′-*β*-FLUORO NUCLEOSIDES)

### Clofarabine

Similar to cladribine, clofarabine (2-chloro-2′-fluoro-2′-deoxy-9-*β*-*D*-arabinofurano syladenine), an ADA-resistant nucleoside analog, is phosphorylated by dCK and then inserted into the DNA chain [37]. Clofarabine effectively inhibited nucleotide reductase and DNA polymerase *α*, leading to DNA strand termination. In late 2004, the FDA approved clofarabine (Clolar®) as the first nucleoside drug for treating lymphoid leukemia in children [38].

**Figure 5.**
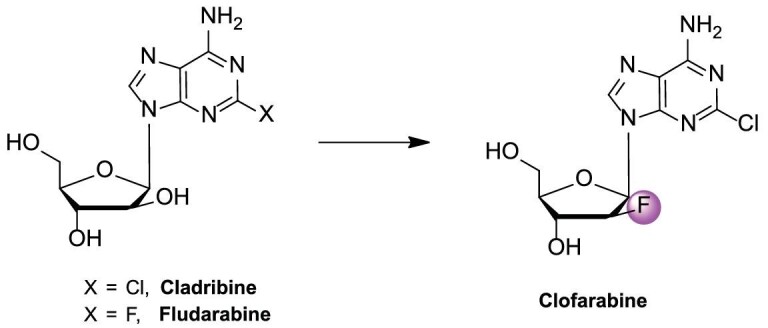
Development pipeline for clofarabine in treating childhood lymphoid leukemia.

Fludarabine and cladribine are first-generation purine-based anti-leukemia drugs, but their glycosidic bonds are easy to break, especially the 2-F adenine produced from the glycosidic bond breakage of fludarabine is toxic [37,38]. The electronegativity of the fluorine atom at the 2′-position effectively resists glycosidic bond breakage [39]. The 2-chloroadenine formed from the breaking of the 2-chloroadenosine was relatively non-toxic. Therefore, clofarabine was designed as a new generation drug for treating leukemia (Fig. 5). After incubation for 72 h, cladribine at 5 nM inhibited growth of K562 cells by 50%. Clofarabine was cleared from the plasma over a period of 1–3 d [40]. A Phase I study established two maximum tolerated doses (MTDs) for clofarabine administered as a 1-h infusion daily over 5 d: 2 mg/m^2^ for solid tumors, with myelosuppression as the dose-limiting toxicity; and 40 mg/m^2^ for acute leukemia, with hepatotoxicity as the dose-limiting toxicity [41]. After the Phase I study in adult leukemia, several Phase II studies were conducted on clofarabine and combination therapies. Consistent drug concentrations were achieved across a wide range of body surface areas with a dose of 52 mg/m^2^. Clofarabine was predominantly bound to albumin in plasma at a rate of 47%. Its primary route of elimination was renal, with 49%–60% of the dose excreted unchanged in urine [42].

### Fiacitabine

Fiacitabine, known as NSC-382097 or FIAC [1-(2-deoxy-2-fluoro-*β*-*D*-arabinofuranosyl)-5-iodocytosine], is a cytidine nucleoside analog. Fiacitabine, synthesized in 1970s by Fox *et al.*, showed antiviral activity against both HSV-1 and HSV-2 (EC_90_ = 0.0025–0.0126 μM) and cytomegalovirus (CMV) (EC_50_ = 0.6 μM) *in vitro* [43,44]. FIAC entered Phase II to study its safety and effectiveness in treating CMV in AIDS patients (ClinicalTrials.gov identifier: NCT00000981) [45]. Unfortunately, the study was terminated due to gastrointestinal side effects experienced by volunteers [46].

**Figure 6.**
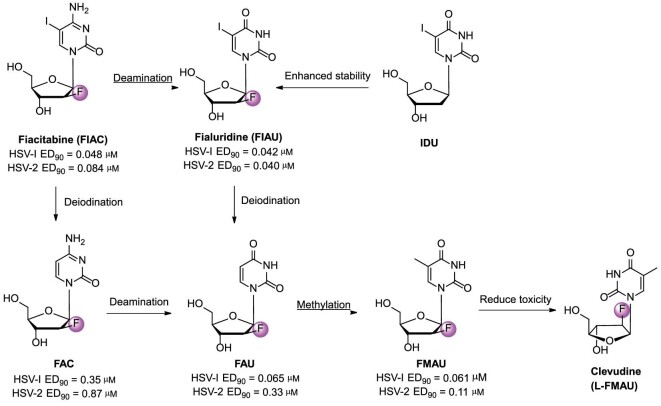
Chemical structures and development pipeline of fiacitabine, fialuridine, and clevudine.

FIAC was mostly metabolized into FAC, FAU, FIAU, and FMAU (Fig. 6), which were then incorporated into DNA [47]. FIAC and its metabolites were more readily phosphorylated by the viral thymidine kinase, selectively inhibiting viral DNA synthesis. FIAC at 10 μM completely inhibited human CMV DNA replication [44]. The potency of the treatment on mice inoculated intracerebrally with HSV-2 strain G was as follows: FMAU >> FIAC ≈ FIAU >> FAC ≈ FAU [48].

The antiviral effects of FIAC were evaluated in duck hepatitis B virus (DHBV)-infected ducks. After intraperitoneal administration for 5 d, FIAC (10 mg/kg/d) induced a transient decrease in DHBV replication in both the serum and liver DHBV DNA level. No toxicity was observed during treatment [49].

### Fialuridine

Fialuridine, known as 1-(2-deoxy-2-fluoro-*β*-*D*-arabinofuranosyl)-5-iodouracil or FIAU (Fig. 6), had good *in vitro* anti-HBV activity (IC_50_ = 0.90 μM) and effectively inhibited HBV replication. Studies showed that FIAU had very low toxicity (CC_50_ = 344 μM) and good therapeutic index (TI = 382.6) [50]. No predictable toxicity was observed in preclinical studies. Additionally, no significant histological or biochemical differences were found when monkeys and dogs were treated with 3 mg/kg/d of FIAC for 90 d and 25 mg/kg/d for 30 d, respectively [46]. FIAU presented a promising alternative for HBV treatment. However, Lilly's Phase II clinical trial of FIAU was halted in June 1993 due to 7 out of 15 subjects dying after 9–13 weeks of treatment [51]. In the previous study, 67 patients did not experience hepatotoxicity after receiving FIAU treatment for 2–4 weeks. However, within 6 months of therapy, three individuals died from liver disease and one from pancreatitis. The clinical incident was re-evaluated, suggesting that it may be due to delayed FIAU toxicity. Further studies elucidated the mechanism of FIAU-induced toxicity. FIAU triphosphate and its metabolite inhibited DNA polymerase γ, leading to decreased mitochondrial DNA levels and structural abnormalities in mitochondria [49,52]. Furthermore, the hepatotoxicity of FIAU in humans was confirmed through studies on chimeric TK-NOG mice with humanized livers [53].

1-(2-Deoxy-*β*-*D*-ribofuranosyl)-5-iodouracil (IDU) is the first antiviral nucleoside, synthesized by Prusoff in 1959 [54], showing promising activity against various orthopoxviruses both *in vitro* and *in vivo* [55]. Unfortunately, its glycosidic bond is extremely unstable, especially in acidic conditions. To address this issue, the structure of IDU was modified by adding a fluorine atom at the 2′-position. The FIAU demonstrated enhanced metabolic stability and potent antiviral activity against various viruses, such as HSV, HBV, VZV, CMN, and EBV [56].

### Clevudine

Clevudine (*L*-FMAU, 1-(2-fluoro-5-methyl-*β*-*L*-arabinosyl uracil, Fig. 6), a non-competitive inhibitor that binds to DNA polymerase with an unnatural L-configuration pyrimidine analog. It was approved in South Korea in 2006 [57]. Clevudine has a long half-life and significantly reduces covalently closed circular DNA, making relapse less likely in patients after treatment is stopped. FMAU, one of the major metabolites of FIAC, had promising inhibitory activity against HSV (ED_90_ = 0.061–0.11 μM), HBV (EC_50_ = 2.0 μM), and EBV (EC_90_ = 0.1 ± 0.02 μM). The clinical use of FMAU was limited due to its myelosuppression and neurotoxicity. However, *L*-FMAU showed promising activity (EC_50_ = 0.1 μM), low toxicity (ID_50_ = 2.0 μM), and excellent selectivity for HBV (SI >2000) [57].

Differently, clevudine is phosphorylated by three enzymes, including cyclosolic thymidine kinase 1, cyclosolic dCK, and mitochondrial deoxyrimidine kinase (also called thymidine kinase 2) [58]. 5′-Triphosphate of *L*-FMAU (*L*-FMAU-TP) not only inhibited HBV DNA polymerase (*K*_i_ = 0.12 μM), but also effectively inhibited DNA-dependent DNA polymerase (EC_50_ = 0.1 μM) [58,59]. In addition, *L*-FMAU did not cause mitochondrial toxicity (CC_50_ >100 μM) [60]. A long half-life (*t*_1/2_ = 44–60 h) and significant reduction of covalently closed circular DNA (cccDNA) were observed in animal models [61].

### Azvudine

The nucleoside-based broad-spectrum antiviral drug azvudine (2′-deoxy-2′-*β*-fluoro-4′-azidocytidine, FNC), is the first-in-class reverse transcriptase (RT)/HIV-1 accessory protein (Vif) dual inhibitor [62]. In 2021, the National Medical Products Administration (NMPA) approved its use to treat HIV-1 infected adult patients [63]. In addition, as an RNA-dependent RNA polymerase (RdRp) inhibitor, FNC has been approved by the NMPA in July 2022 and by the Ministry of Health of the Russian Federation in February 2023 to treat patients with COVID-19 infection [64,65]. The clinical trial for azvudine, which aims to prevent COVID-19 infection after exposure, has been approved to take place in the Philippines on December 22, 2022 (Clinical Trial Reference Number: 2022-CT0714).

**Figure 7.**
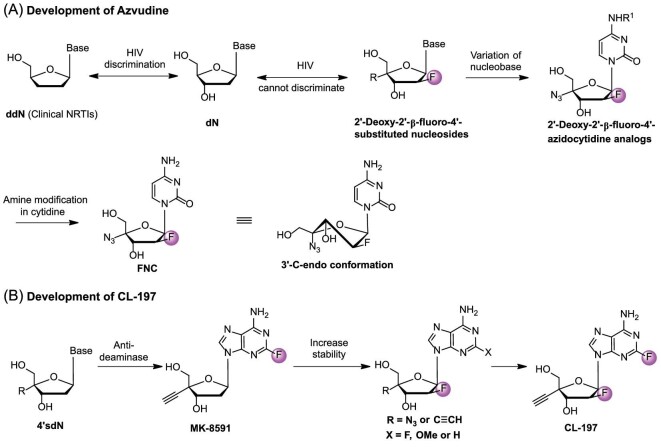
(A) Development of azvudine for the treatment of HIV and COVID-19 infection. (B) Development of CL-197 for the treatment of HIV infection.

Nucleoside reverse transcriptase inhibitors (NRTIs) are part of the 2′,3′-dideoxynucleoside (ddN) family. They play a crucial role in stopping the biosynthesis of proviral DNA catalyzed by RT. As HIV-1 mutants appeared, ddNs were distinguished from 2′-deoxynucleosides (dNs). This led to their obstruction in the active center of RT or detachment from the end of proviral DNA. The presence of a 3′−OH group in NRTIs is vital for inhibiting HIV variants. A series of 2′-deoxy-2′-*β*-fluoro-4′-substituted nucleosides (R = N_3_, CN, Me, ethynyl, substituted triazole; nucleobase = uracil, thymine, purine) synthesized by our team have all functional groups of dNs, which made it difficult to discriminate from natural dNs [66,67]. The stability of glycosidic bonds was enhanced by the introduction of 2′-fluorine, particularly in acidic conditions. In SAR studies on cytidine nucleoside derivatives with 4′-azide, it was found that adding substituents to the amine decreased antiviral activity [68]. The addition of a 4′-azido group altered the conformation of the furanose ring to a 3′-C-*endo* conformation, resulting in significantly enhanced activity and making it effective against resistant HIV strains (Fig. 7A).

The hydrochloride form of azvudine demonstrated high effectiveness against both wild-type and drug-resistant viral strains (Table 3). Specifically, for the wild-type and HIV-resistant strains, the EC_50_ values were as follows: HIV-1 (wild-type) at 0.13 nM, NL4-3 (wild-type) at 0.086 nM, NL4-3 (K101E) at 0.15 nM, and HIV (RTMDR) at 0.11 nM. In the case of resistant strains like 7324–1, 7324–4, 7303–3, 35764–2, and 53252–1, FNC displayed potent inhibitory activity in the nanomolar range with EC_50_ values ranging from 0.42 to 0.735 nM. Notably, azvudine exhibited a remarkably low EC_50_ value of only 0.063 nM against vesicular stomatitis virus glycoprotein (VSVG) pseudotyped single-cycle infectious virus (HIV-luc/VSV-G), as detailed in Table 3 [69]. Cellular kinases converted FNC into FNC-TP, which then selectively entered target cells. The 50% reduction of HIV-1 prophylaxis data (T_50%-prevention_ of 0.1 and 10 nM = 68.6 and 123.1 h, respectively) indicated that FNC had a long-lasting preventive effect against HIV-1 infection [62].

**Table 3.** Anti-HIV activity of azvudine

Virus type	EC_50_ (nM)	Virus type	EC_50_ (nM)
HIV-1 (wild-type)	0.13	4755–5 (M41L, D67N, L210W, T215Y, M184V, T69D, E44D, V118I)	>40 000
NL4-3 (wild-type)	0.086	6463–13 (M41L, D67N, L210W, T215Y, M184V, V118I)	>40 000
NL4-3 (K101E)	0.15	29129–2 (M41L, D67N, L210W, T215Y, M184V)	>40 000
HIV (RTMDR)	0.11	1617–1 (K70G, M184 V, T69K, V75I, F77L, F116Y, Q151M)	32.2
7324–1 (M41L, D67N, K70R, T215F, K219E,T69N)	0.595	7303–3 (M41L, D67N, L210W, T215Y, T69D, E44D, V118I)	0.56
7324–4 (M41L, K70R, T215F, K219E)	0.735	35764–2 (V75I, F77L, F116Y, Q151M)	0.42
10076–4 (M41L, T215Y, M184V)	>40 000	56252–1 (K70R, V75I, F77L, F116Y, Q151M, K65R)	0.525
7295–1 (D67N, K70R, T215F, K219Q, M184V, T69N)	>40 000	HIV-luc/VSV-G	0.063

FNC was phosphorylated to create FNC-TP, which then joined the chain in the SARS-CoV-2 synthesis process, halting RNA replication. FNC exhibited potent anti-SARS-CoV-2 effects (EC_50_ = 1.2–4.3 μM) with good selectivity (SI = 15–83) [70]. In a Phase Ⅲ clinical trial, FNC significantly reduced the time for symptoms to improve in patients with moderate SARS-CoV-2 infection. FNC effectively inhibited virus mutations (Alpha, Beta, Delta, and Omicron) and shortened the virus clearance time to ∼5 d. On average, there was a 1.56 log10 decrease in viral load after 5 d of treatment [64]. PK studies in rhesus macaques revealed that azvudine was rapidly absorbed in plasma and entered the PBMCs, forming the active nucleoside triphosphate FNC-TP with intracellular half-lives of 133.15 and 112.40 h, respectively, after dosing at 1 and 6 mg/kg [62]. FNC showed a chemo-immune behavior that could be effective against viruses like AIDS and COVID-19, which attack the immune system, indicating FNC's immune-targeting properties, promoting thymus-homing and immunity in animal models [70]. The organ distribution studies revealed that FNC was detectable in all organs, with the highest levels observed in the thymus and spleen within the first 2 h. Notably, only the thymus showed presence of FNC triphosphate (FNC-TP) [70]. After being given 0.5 mg/kg of FNC by gavage in Sprague–Dawley rats, the FNC-TP was primarily distributed in the thymus, with peak concentration reached at 7–12 h [62]. This may explain the high efficacy of FNC in treating COVID-19 and AIDS.

In addition to its anti-HIV and anti–SARS-CoV-2 effects, FNC also demonstrated broad inhibitory activity against various viruses including HBV, HCV, and enteroviruses such as EV71, CA16, CA6, EVD68, and CVB3 [71]. Notably, FNC exhibited a potent inhibitory activity on HBV antigen secretion in HBV-transfected HepG2.2.15 cells (HBsAg EC_50_ = 0.037 μM, HBeAg EC_50_ = 0.044 μM) and reduced the HBV DNA level by >90%. In ducks infected with DHBV and treated with FNC, suppression of DHBV replication and histopathological improvement were observed. FNC exhibited inhibitory activity against both wild-type and lamivudine-resistant HBV clinical isolates (EC_50_ = 0.12 ± 0.01 nM, 0.27 ± 0.01 nM, respectively) [72].

Due to the potential for both cell cycle arrest and the suppression of retrotransposons/HERVs, FNC could cause termination of the synthesized nucleic acid chain and thus demonstrated good anti-cancer activity [72]. Modulating the immune system may be another crucial mechanism of FNC in fighting cancer, and suppressing retroviral activity can enhance its immunotherapeutic effects. The anti-cancer effects of FNC have already been shown in non-small cell lung cancer cells (NSCLCs), non-Hodgkin lymphomas [73], diffuse large B-cell lymphoma (DLBCL) [74], transformed follicular lymphoma, and acute myeloid leukemia (Table 4) [74]. The *in vivo* antitumor activity of FNC was assessed on SCID/Beige mice bearing JeKo-1 tumors at doses of 1, 2, and 3 mg/kg. The inhibitory rates were 37.9%, 75.8%, and 82.1%, respectively. At the end of the experiment, compared with the control group (tumor volume was 1857.73 ± 326.51 mm^3^), the tumor volume in the groups receiving FNC (1, 2, and 3 mg/kg) was 1089.35 ± 267.14, 452.65 ± 96.38, and 274.40 ± 77.26 mm^3^, respectively. The body weight of the low- and medium-dose FNC groups did not decrease, and histopathological examination showed no signs of organ tissue toxicity in the liver and kidney [75]. Similar anticancer effects were also observed in mouse xenograft models of hepatocarcinoma (H22), sarcoma (S180), and gastric carcinoma (SGC7901). These findings demonstrate that FNC significantly inhibited tumor growth with minimal toxicity [74].

**Table 4.** Anti-cancer activity of FNC.

Tumor type	Tumor cell line	IC_50_ (μM)
NSCLC	H460	0.267
	A549	1.22
Non-Hodgkin lymphomas	Raji	0.2
	JeKo-1	0.097
	Granta-519	0.95
Acute myeloid leukemia	HL-60	3.3
Transformed follicular lymphoma	SUDHL-6	4.55
DLBCL	RL	1.74

**Figure 8.**
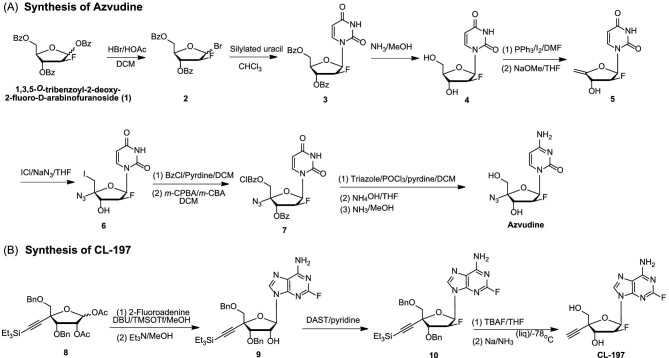
Synthesis of azvudine (A) and CL-197 (B).

The synthesis of azvudine is shown in Fig. 8A. 1,3,5-*O*-tribenzoyl-2-deoxy-2-fluoro-*D*-arabinofuranoside was brominated at the 1-position to yield *α*-bromide (**2**). The α-bromide (**2**) reacted with silylated uracil in a glycosylation process to produce *β*-nucleoside (**3**). Treating compound **3** with methanolic ammonia resulted in high-yield nucleoside **4**. Subsequent iodination and elimination reactions led to the formation of 4′-methylene-nucleoside (**5**). Compound **5** was then treated with ICl/NaN_3_ to generate 4′-azido-nucleoside (**6**). The substitution of the 5′-iodine on compound **6** with *m*-chlorobenzoyl and protection of the 3′-OH group by a benzoyl group gave compound **7**, and subsequent amination and deprotection steps ultimately yielded azvudine [69].

### CL-197

Due to the addition of 4′-ethynyl and 2-fluorine atoms, MK-8591 (Fig. 8) demonstrated enhanced efficiency in being integrated into the new DNA strand and resistance to purine deaminase. This led to potent anti-HIV activity and long-lasting effects [76]. Nonetheless, the glycosidic bond was susceptible to breakage, leading to the formation of toxic 2-F adenine [37]. Our group has always focused on studying 4′-modified nucleoside analogs. Given the approval of the 4-substituted NA (azvudine) for treating HIV-1 and COVID-19, we have discovered a new anti-HIV agent. The 2′-fluorinated purine NAs with an ethynyl or Azido group at the 4′ position show significant anti–HIV-1 activity at nanomolar levels [10].

In 2014, our group reported a series of 2′-fluorine-4′-substituted NAs, of which CL-197 (4′-ethynyl-2-fluoro-2′-fluoro-2′-deoxyadenosine, EFFdA) showed potent anti-viral activities [77]. 2′-Fluorine increased the stability of the glycosidic bond and reduced the formation of toxic 2′-F adenine (Fig. 7B) [39]. CL-197 inhibited reverse transcription by simulating endogenous purine nucleotides and showed long-term effect in the treatment of HIV infection [10]. CL-197 effectively inhibited HIV replication and is expected to be taken orally once a week. PK studies showed that after 24 h of intragastric administration of CL-197 (6 mg/kg) in macaque, CL-197 cannot be detected in plasma, but the active CL-197-TP in HIV target cells and peripheral blood mononuclear cells of macaque was still >0.11 nM after 7 d of gavage, while the half-life of the drug in the target cells was more than 72 h. However, when the reference compound was administered at a dose of 20 mg/kg for 24 h, neither 3TC nor its active component, 3TC-TP, were detected in the plasma and PBMCs of rhesus monkeys [67]. The major advantage of CL-197 is its long-acting characteristics after oral dosing that allows oral administration once a week [67]. Thus, CL-197 could serve as a long-lasting medication for the prevention and treatment of AIDS. The prolonged action of CL-197 is likely due to the addition of 2-fluorine and improved metabolic stability from including 4′-ethynyl. Preclinical studies concluded in December 2021. CL-197, a long-acting nucleoside for AIDS treatment, will soon undergo clinical trials with adult HIV-1 patients (Registration No.: CXHL2200529).

The glycosylation of protected sugar **8** with 2-fluoroadenine using 1,8-diazabicyclo[5.4.0]undec-7-ene (DBU) and trimethylsllytrifluoromethanesulphonate (TMSOTf), followed by deacetylation with triethylamine, produced compound **9**. The hydroxyl group at the 2′ position of compound **9** was converted to a fluorine atom using diethylaminosulfur trifluoride (DAST) to yield 2-fluoronucleoside **10** with an inverted configuration. Subsequent removal of the *t*-butyldimethylsilyl (TBS) group with tetrabutylammonium fluoride (TBAF) and benzyl group *via* Birch reaction resulted in the formation of CL-197 (Fig. 8B) [10].

## NUCLEOSIDES CONTAINING TWO FLUORINE ATOMS AT C-2′

### Gemcitabine

Since the discovery of cytarabine (ara-C), there have been extensive structural modifications made around ara-C. Due to the unique properties of fluorine, fluorinated NAs were found to have varied bioactivities while only undergoing minor changes in shape. Among these compounds, the 2′,2′-fluorine NAs have exhibited potent antitumor activities. To increase the oral bioavailability of cytarabine and enhance the NAs transport through membranes, replacement of −OH with fluorine in the nucleotide ribose ring was performed. Replacing the −OH group at the 2′-carbon with a single fluorine atom in cytarabine led to the formation of 2′-deoxy-2′-fluorocytidine (F-ara-C). This modification increased its cytotoxicity by 10-fold, but the compound exhibited limited antitumor activity *in vivo* [78]. Subsequent alterations based on F-ara-C gave potent antiviral nucleosides (such as FIAC, FEAU, and FMAU).

Replacement of both hydrogen atoms at C-2′ with fluorine may further increase cytotoxicity and bioavailability of F-ara-C. Gemcitabine (2′-deoxy-2′,2′-fluorocytidine, dFdC) is one of the 2′-difluoro substituted deoxycytidine analogs [79]. Gemcitabine was ineffective as an antiviral in host cells. However, due to its structural and metabolic similarities to cytarabine, it exhibited unique anti-cancer properties by effectively inhibiting the growth of a wide range of solid tumors and hematological malignancies.

**Figure 9.**
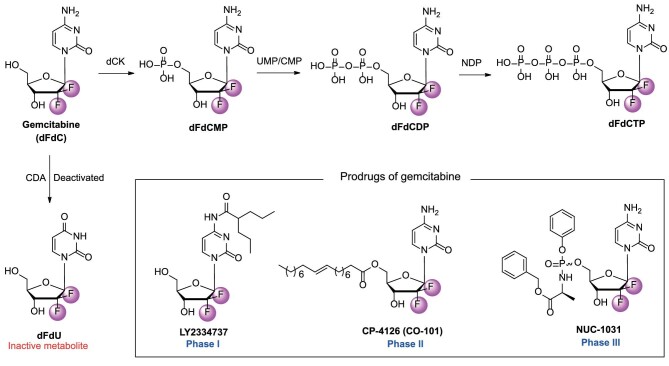
The structures of gemcitabine, mono-, di-, triphosphorylated gemcitabines, and its prodrugs.

The effectiveness of gemcitabine in fighting tumors is linked to its complex metabolism, which includes self-potentiation and the inhibition of DNA synthesis by its diphosphate and triphosphate nucleosides. Like other nucleoside analogs, gemcitabine undergoes stepwise phosphorylation to be incorporated into DNA, ultimately halting DNA synthesis in cancer cells (Fig. 9). The first phosphorylation step of gemcitabine is catalyzed by dCK, which is also the crucial phosphorylation step like that of ara-C, to give the monophosphate form (dFdC-MP) [80]. Then the second phosphorylation stage of gemcitabine is accomplished by UMP/CMP kinase to produce the diphosphate form (dFdC-DP), which is responsible for its self-potentiation mechanisms [81]. dFdC-DP inhibits ribonucleotide reductase (RNR), which decreases the conversion of cytidine diphosphate (CDP) to 2′-deoxycytidine diphosphate (dCDP), leading to lower levels of 2′-deoxycytidine triphosphate (dCTP). Gemcitabine triphosphate (dFdC-TP) competes with dCTP for DNA integration. This decrease in intracellular dCTP concentration boosts the integration of gemcitabine triphosphate into DNA, a process known as self-potentiation [82].

After gemcitabine is translated into its active form, dFdC-TP, it can interact with DNA polymerases. *In vitro* DNA primer extension assays showed that dFdC-TP competes with dCTP for incorporation at the C sites of the DNA strand being synthesized, the *K*_i_ values for dFdC-TP were 11.2 μM and 14.4 μM for DNA polymerase alpha and polymerase epsilon, respectively [83]. After being incorporated into the DNA chain, gemcitabine halted further elongation by acting as an abnormal nucleotide, leading to drug-induced cell death or apoptosis. Gemcitabine killed cells in the S-Phase undergoing DNA and RNA synthesis, and it also blocked cell progression through the G1/S-Phase transition [84]. Moreover, dFdC permeated the membrane 65% faster than ara-C, and intracellular elimination of dFdC-TP lasted longer compared to ara-C, which is biphasic with *t*_1/2_ alpha = 3.9 h and *t*_1/2_ beta >16 h [85]. Studies showed that gemcitabine had a broad-spectrum antitumor activity against cancers such as NSCLC, ovarian, breast, bladder, pancreatic, and colon cancers [86]. It is administered once weekly for three weeks as a parenteral formulation, given through a 30-min intravenous infusion at a dosage of 1000–1250 mg/m^2^ [87]. Gemcitabine and its hydrochloride (Gemzar®) have been approved for cancer treatment.

### LY2334737

Gemcitabine, despite being an effective chemotherapeutic drug, faces challenges that hinder its long-term use in cancer treatment. The main obstacles include inherent and acquired resistance mechanisms. One notable challenge is the inactive metabolite 2′,2′-difluoro-2′-deoxyuridine (dFdU), which is caused by the deactivating enzyme cytidine deaminase (CDA) found in the human liver and kidney [88]. The prodrug strategy is a practical approach in medicinal chemistry that aims to improve drug stability, enhance PD/PK properties, and eliminate side effects. The design of gemcitabine prodrugs primarily focused on its 4-amino group and the 5′-hydroxyl group. LY2334737, CP-4126, and NUC-1031 are representative gemcitabine prodrugs (Fig. 9).

The 4-amino group on the cytidine ring suffers from CDA metabolism, which diminishes the efficacy of gemcitabine. Direct modification at the 4-amino group could solve this issue. In 2009, Bender *et al*. modified the 4-amino position of the gemcitabine cytosine with valproic acid to develop an orally effective gemcitabine prodrug LY2334737 (Fig. 9) [89]. LY2334737 is orally bioavailable with remarkable enzymatic stability. In CD-1 mice and human small intestine homogenates, the hydrolysis rate of LY2334737 was slow (<10 pmol/min/mg). The hydrolysis rate in human S9 was relatively slow at 27 pmol/min/mg and in mouse S9 it was 11 pmol/min/mg. These results suggested that LY2334737 was absorbed orally as the intact prodrug. In a PK study conducted on CD-1 mice, gemcitabine was administered orally at a dose of 14.3 mg/kg. The resulting systemic exposure for gemcitabine was good (AUC = 778 ng·h/mL, with a *C*_max_ of 373 ng/mL). This is in comparison to the AUC value of 536 ng·h/mL and *C*_max_ of 535 ng/mL observed under the same oral dose of gemcitabine. The *T*_max_ of LY2334737 was 1 h, which evidenced that it was more stable than gemcitabine (*T*_max_ = 0.5 h). Moreover, the ratio of dFdU to gemcitabine in mice treated with LY2334737 was less than half compared to mice treated with an equal dose of gemcitabine. This demonstrates that N^4^-modification is a viable strategy for discovering new nucleoside-based prodrugs.

An *in vivo* study showed that tumor volume decreased by 67.1% with a 7.55 mg/kg dose of LY2334737 (p.o., qd × 14), similar to the reduction (71.5%) seen with gemcitabine at a dose of 160 mg/kg (i.p., q3d × 4). The results indicated that orally administering LY2334737 showed significant antitumor activity in HCT-116 human colon tumor xenografts. LY2334737, a potent oral anticancer prodrug of gemcitabine, is currently undergoing clinical trials. Multiple Phase I clinical trials have been conducted using LY2334737 to treat advanced and/or metastatic solid tumors either alone or in combination with other anticancer drugs, with the MTD of 40 mg/d. The metabolite dFdU accumulated with an accumulation index of 4.3 (CV: 20%) [90]. In addition, LY2334737 exhibited antiviral activity against enterovirus infection [91].

### CP-4126

In addition to the 4-amino group, the 5′-hydroxyl group is also a suitable site for designing gemcitabine ester prodrugs. The high hydrophilicity of NAs, including gemcitabine, limits their ability to pass through cell membranes by passive diffusion [92]. Improving lipid solubility could alter nucleoside transport activity. Thus, a fatty acid ester derivative of gemcitabine CP-4126 (CO-101, Fig. 9) was synthesized by coupling elaidic acid (trans-9-octadecenoic fatty acid) to the 5′ position on the sugar moiety in CP-4126. The fatty acid derivative CP-4126 had the potential to effectively transport across cell membranes and prevented drug resistance [92]. Besides, with the fatty acid chain, CP-4126 effectively protected gemcitabine from deamination [92]. The *in vitro* IC_50_ values of CP-4126 in solid tumor cells were similar to gemcitabine. Administered via intraperitoneal and oral routes, CP-4126 showed efficacy against human tumor xenografts. In the EKVX NSCLC xenograft model, both gemcitabine and CP-4126 demonstrated equal effectiveness, with T/C values of 3.4% and 2.9%, respectively [92]. In the CRL-1435 prostate cancer xenograft model, both gemcitabine and CP-4126 showed activity with specific growth delay factors (SGDs) of 2.9 and 3.2, respectively. Gemcitabine was poorly tolerated in the MiaPaCa-2 and PANC-1 pancreas xenografts, leading to toxic deaths of 7 out of 7 and 4 out of 9, respectively [92]. The oral bioavailability of CP-4126 in two dogs was found to be undetectable in plasma after oral administration but detectable after intravenous (*i.v.*) administration. The levels of dFdC were higher compared to those observed after *i.v.* administration.

CP-4126 was approved for a Phase I clinical trial as an antitumor medication in 2008 (ClinicalTrials.gov identifier: NCT 00778128). The study was to determine the recommended dose and MTD of CP-4126, establish PK characteristics and safety profile, and preliminarily assess its antitumor activity [93]. CP-4126 was well tolerated with a toxicity profile comparable to gemcitabine. The MTD and recommended Phase II dose were 1250 mg/m^2^ [94]. The initial half-life (*t*_1/2α_) of dFdC formed from CP-4126 (*t*_1/2α_ = 0.25 h) was twice that of dFdC formed from gemcitabine (*t*_1/2α_ = 0.12 h), as dFdC is continuously produced from CP-4126 in plasma [94]. In the subsequent Phase II study, CP-4126 was evaluated for its efficacy in treating metastatic pancreatic cancer, a gemcitabine refractory disease with non-expressing tumors. This study used a new trial design with biomarker selection and a Simon II stage design. The trial would stop in stage I if fewer than three patients failed to achieve disease control (complete response (CR) + partial response (PR) + stable disease (SD)) [95]. Unfortunately, the study was halted after the first stage because it did not meet the endpoint. Only two out of 18 patients achieved disease control [95].

### NUC-1031

CP-4126 showed metabolic improvements by attaching a fatty acid chain to the ribose 5′-position. However, the fragility of the 5′-ester prodrugs in releasing free gemcitabine prevented it from demonstrating superiority over gemcitabine in clinical trials. To overcome this issue, in 2014, Slusarczyk *et al*. used the ProTide technology to design a series of gemcitabine phosphoramidate prodrugs. NUC-1031 is the most potent gemcitabine phosphoramidate prodrug which has entered clinical studies for cancer treatment (Fig. 9). *In vitro* cytotoxicity of NUC-1031 against human pancreas adenocarcinoma BxPC-3 and MiaPaCa-2 cell lines were much higher than that of gemcitabine (2- to 4-fold increase) with IC_50_s of 0.44 and 0.15 μM, respectively [96]. The improved activity was mainly due to increased intracellular delivery, because NUC-1031 could enter tumor cells without the help of hENT1 transporter proteins [97]. As the protective motif leaves NUC-1031 by ester hydrolysis, spontaneous cyclization, nucleophilic attack of the carboxylate residue, the active gemcitabine monophosphate was generated. NUC-1031 was stable with *t*_1/2_ of 139 min in human hepatocytes and 18% still remained in liver microsomes after 1 h [96]. After first administration of NUC-1031 (0.076 mmol/kg, i.p.), there was a significantly greater reduction in tumor volume than for gemcitabine on Day 7 [96]. Early Phase I studies showed that NUC-1031 was well tolerated and had favorable PK profiles [97]. The Phase II and III studies of NUC-1031 were terminated because it is uncertain whether the primary objective of improving overall survival can be achieved in this heavily pre-treated population with significant co-morbidities.

### Rovafovir etalafenamide

**Figure 10.**
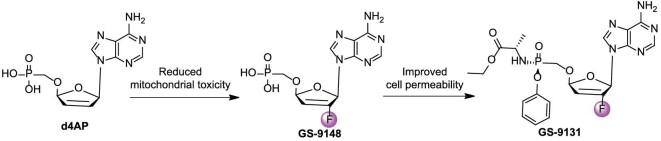
Development pipeline of GS-9131 for the treatment of HIV infection.

d4AP showed superior activity against both wild-type (EC_50_ = 2.1 ± 1 μM) and NRTI-resistant HIV-1 strains (EC_50_ fold change <2.9), but the replication of mtRNA was easily interfered with by d4AP, resulting in mitochondrial toxicity (MTC_50_ = 3.6 ± 1.5 μM). The GS-9148 produced by the introduction of 2′-F showed potency against wild-type (EC_50_ = 10.6 ± 2.4 μM) and drug-resistant HIV-1 strains (EC_50_ fold change <4.3), and significantly reduced mitochondrial toxicity (MTC_50_ >300 μM) [98]. GS-9131 had more potential in activating PBMCs (EC_50_ = 3.7 nM) than in MT-2 cells (EC_50_ = 150 nM), indicating that the introduction of prodrug moiety greatly improved cell permeability (Fig. 10) [98].

Rovafovir etalafenamide (GS-9131) is an oral NRTI derived from adenine nucleoside GS-9148 developed by Gilead Sciences [99]. GS-9131 had potent inhibitory activity against different subtypes of HIV-1 (UG-92–031 subtype A, B940374 subtype B, LJM subtype B, BR-92–025 subtype C, and UG-92–024 subtype D) and HIV-2 (CDD77618 subtype A, CDD310248 subtype A, and CDD310319 subtype B), with EC_50_ values of 23–68 nM and 390–650 nM, respectively [98]. GS-9131 did not significantly affect the activity of RT mutations K65R, L74V, M184V, or their combinations (EC_50_ fold change <1) [100]. Unfortunately, the Phase II clinical trial of GS-9131 was terminated because it did not meet the targeted antiviral response (ClinicalTrials.gov identifier: NCT03472326).

## CONCLUSIONS

The fluorine atom plays a crucial role in medicinal chemistry by significantly enhancing the PK and PD properties of bioactive compounds. To date, over 30 fluorinated nucleosides have advanced to clinical trials or been approved as anticancer, antiviral, or antibacterial drugs. Among them, 12 contain fluorinated nucleobases and 25 are analogs with fluorinated ribose rings. The rapid advancement of fluorinated NAs is closely dependent on the development of methodology for introducing fluorine substituents in NAs, which may largely expand the implications of fluorinated NAs. Two main approaches have been reported to synthesize 2′-fluorinated nucleosides, including (1) anhydronucleoside fluorination with HF or KF, and (2) arabinonucleoside fluorination *via* a sulfonate intermediate using DAST or with TBAF. Fluorinated NAs have been extensively developed to address the urgent challenges of repurposing drugs during the COVID-19 pandemic (such as azvudine). However, there is still significant potential for the development of novel agents that possess broad-spectrum antiviral activity and anticancer properties. Fluorinating nucleosides and/or nucleotides offers valuable practical and versatile insights for the rapid preparation of broad-spectrum agents. Besides, various modifications of oligonucleotides could also benefit from the discovered fluorinated NAs. Chemical modification, particularly the prodrug strategy, is one of the most effective ways to modulate PK profiles and improve their efficacy. That is always attributed to the unique properties of the fluorine atom, such as small size, strong electronegativity, and lipophilicity. In this Review, we provide a comprehensive summary of 2′-fluorinated nucleoside analogs that have been approved or are currently undergoing clinical evaluation for anticancer and antiviral treatment. The primary aim is to emphasize the significant impact of fluorinated NA medications and offer perspectives on upcoming NAs. The design principle, main SAR studies, and associated metabolism pathways discussed may also apply to other fluorinated nucleosides beyond just 2′-fluorinated ones.
